# Pain and Diet: A summary of the evidence for the role of diet modification in chronic pain

**DOI:** 10.1177/20494637231203856

**Published:** 2023-09-30

**Authors:** David Cameron, Joanna Harrison, Shiva Tripathi, James Hill

**Affiliations:** 1 Lancashire Teaching Hospitals NHS Trust; 2 Synthesis, Economic Evaluation and Decision Science Group (SEEDS), University of Central Lancashire

**Keywords:** chronic pain, dietary change, dietary ingredients, PUFA, vegetarian

## Abstract

Chronic pain constitutes a significant burden to patients and healthcare systems. For many patients the only option is to attempt to manage their pain within their daily lives. Here we review evidence provided by three systematic reviews for the effect of diet and diet supplements on patients’ experience of chronic pain.

## Introduction

Chronic pain is defined as pain that persists or recurs for more than three months^1^. Chronic pain is common in the United Kingdom, affecting up to 30-50% of the population^2^, is self-reported in all adult age groups, ethnicities, and socioeconomic backgrounds, and is seen to increase in prevalence in later years of life^3^. Chronic pain is associated with reduced physical and mental health-related quality of life for patients and with negative impacts on social relationships and interactions in the workplace^4^. Healthcare resources feel a significant burden from chronic pain, with hundreds of millions of pounds spent annually by the NHS on pain conditions^5,6^.

The National Institute for Health and Care Excellence (NICE) recommends several evidence-based approaches for clinicians managing chronic pain in adults^2^. These include exercise programmes, psychological therapy, and acupuncture alongside antidepressant use if justified^2^. Due to limited evidence of efficacy, the use of benzodiazepines, non-steroidal anti-inflammatories, paracetamol, ketamine, opioids, local anaesthetics or corticosteroids is not recommended in such scenarios. NICE recommend research into alternative pain management strategies such as further types of psychological therapies, manual therapies and social interventions (e.g exercise and nutrition assessment).

It is known that chronic pain increases with Body Mass Index (BMI)^3^. A higher BMI is also associated with an increased risk of back pain and musculoskeletal pain^7,8^. Several systematic reviews have been published on different elements of dietary interventions for managing chronic pain^9–12^. It is now timely to provide an overview of this evidence to help guide recommendations for patients. The three systematic reviews discussed in this commentary^9–11^ were selected on the quality of evidence reported for relevant pain outcomes (moderate to high).

## Aims

This commentary aims to explore and critically appraise three systematic reviews by Crawford et al. ^9^, Field et al.^10^ and Prego-Dominguez et al.^11^ and expand upon the findings in the context to clinical practice (see Supplementary file 1 for methods, full results and critical appraisal).

## Commentary

The findings from the three reviews suggest the following: a) whole food dietary change has a small yet positive effect on chronic pain based on average to good quality studies, noticeably so for vegetarian/vegan or Mediterranean diets and single food changes (adding plant-based food containing bioactive compounds), b) PUFA supplementation has a small but positive effect on reducing chronic pain based on mostly high-quality studies, and noticeably so for Omega 3 fish oil and when given for less than 3 months at a low dosage (daily intake ≤ 1.35g), c) Dietary ingredients of capsaicin, ginger, and rosehip have a small to medium sized positive effect on chronic pain based on moderate to high quality evidence (see Table 1 for findings).

As there appears to be initial evidence for the beneficial effects of some diets & dietary supplements for use in chronic musculoskeletal pain, the results from these reviews present interesting possibilities for applications in practice. The attraction of these possible benefits is that supplementation is widely available (especially in the UK) without the need for a prescription. Capsaicin, Ginger, Rosehip, and Omega 3 are available as dietary preparations in most supermarkets or supplement shops. Where appropriate, and without evidence of negative health impacts, this is likely to sit well alongside other interventions planned for patients with chronic pain. For specific diets (e.g. Vegetarian, Vegan) this would have to be considered by individual patients as it may reflect a significant change in lifestyle that they are unwilling or unable to adopt. Consideration must additionally be given to the fact that dietary supplementation has the potential to represent a significant financial cost to patients. Chronic pain is more prevalent in areas of greater socioeconomic deprivation^3^ and it is acknowledged that this must be taken into account when educating patients on potential benefits.

Therapeutic patient education (learning competencies, adapting behaviours) which is both multi-disciplinary and multi-dimensional has previously been described as having a beneficial impact for patients with chronic diseases^16^, and a similar approach may be appropriate in chronic pain. NICE guidance for practitioners on approaches to patient education in the prevention of obesity^17^ remains relevant for dietary advice with chronic pain. The guidance highlights the importance of good communication between health professionals and patients and adds that advice should be non-discriminatory, culturally appropriate, written (where appropriate) and tailored to patients’ needs.

Patient education that includes verbal teaching with written material, audio or videotapes may also be beneficial^18^. Clinicians treating chronic pain will be aware of the unique and subjective impact pain has on each patient and their lifestyle. As such, pain specialists should employ personalised recommendations on possible dietary changes based on individualised discussion for each patient, rather than rely on a set methodology.

Our commentary suggests that there is a gap in the research exploring the development of chronic pain and different types of diets. Despite the moderate to high quality evidence available, considerable variability exists in studies of this nature and more work is needed to identify dietary interventions that are effective. Future interventions should be well defined to determine the factors that influence the intervention such as: vegan vs vegetarian vs non-vegetarian (white meat vs red meat), amount of salt, sugar, fat content, information provided, and mode/frequency of intake. Future research should also ensure to use standard reporting frameworks such as the template for intervention description and replication^19^. Furthermore, rather than using unidimensional pain assessments like the VAS and the Numeric Rating Scale, multi-dimensional pain assessment tools should be used to assess dietary interventions. Outcome measures should also consider the biopsychosocial context including physiological measures: physical functioning, development of chronic illness (diabetes, Cardiovascular, fibromyalgia), psychological measures: (anxiety, depression, catastrophising) and social measures (family structure, wealth, eating habits, education, employment and benefits). A longitudinal observational study (20-30 years) of young adults combining the above outcome measures would help to inform how diet can be changed to avoid or minimise certain pain conditions in the long term. In addition, future research should explore what are the mediating factors of the supplements (e.g. curcuma and its anti-inflammatory effect, specific diets and reduction in BMI).

## Supplementary Material

Supplementary file 1

## Figures and Tables

**Table 1:** Findings of Crawford et al. ^9^, Field et al.^10^ and Prego-Dominguez et al.^11^.

Estimates of effect for dietary interventions on pain reduction				
**Intervention** * statistically significant	**Number and type of trial**	**Estimate of effect** SMD (95%CI), *p* value, *I^2^.*	**Interpretation of effect and heterogeneity**	**Quality Assessment of included studies** (summary)
**Crawford et al.^9^**				
Capsaicin*	8 RCTs	SMD -0.56 (-0.72 to -0.39), *p* < 0.00001 *I^2^* = 26%	Medium significant effect, heterogeneity might not be important	GRADE: High
Ginger*	5 RCTs	SMD = -0.30 (-0.09 to -0.50), *p*=0.005, *I^2^* = 27%	Small significant effect, heterogeneity might not be important	GRADE: Moderate
Rose Hip*	3 RCTs	SMD =0.37 (0.13 to 0.6), *p* =0.0019,*I^2^* = 0%	Small significant effect, zero heterogeneity reported	GRADE: Moderate
Boswelia*	6 RCTs	SMD= -3.34 (-4.86 to -1.82), *p* < 0.0001, *I^2^*=94%	Large significant effect, considerable heterogeneity	GRADE: Very Low
Curcuma*	3 RCTs	SMD =-1.05 (-1.68 to -0.02), *p=*0.001 *I^2^*=65%	Large significant effect, substantial heterogeneity	GRADE: Low to Very Low
Vitamin D*	8 RCTs	SMD= -0.55 (-0.99 to-0.11), *p*=0.001 *I^2^*=92%	Medium significant effect, considerable heterogeneity	GRADE: Low
Pycnogenol *	3 RCTs	SMD =-0.75, (-1.30 to -0.20), *p=0.007, I^2^*=76%	Medium significant effect, considerable heterogeneity	GRADE: Low
Avocado Soybean Unsaponifiables	5 RCTs	SMD =-0.34 (-0.72 to 0.03), *p*=0.07, *I^2^* = 88%	Small non-significant effect, considerable heterogeneity	GRADE: Moderate to Low
Glucosamine plus chondroitin	Not reported	SMD= -0.27, (-0.47, -0.06)	Small effect, significance level unclear, heterogeneity not reported	Not reported
Collagen derivatives	4 RCTs	SMD =-0.01 (-0.32 to 0.34), *p* = 0.93, *I^2^*= 76%	Small non-significant effect, considerable heterogeneity	GRADE: Low
Willow bark extract	3 RCTs	SMD= -0.29 (-0.57 to 0.00), *p*=0.05, *I^2^* =0%	Small non-significant effect, zero heterogeneity reported	GRADE: Low
Polyunsaturated Fatty Acids reported in Prego-Dominguez et al.^11^				
**Field et al.^10^**				
Whole foods overall(combined dietary groups)*	22 RCTS, 1 Case Controlled study (25 intervention groups)	SMD= -0.44(-0.63 to -0.24), *p* <0.0001,*I^2^* =62%	Small significant effect, substantial heterogeneity	Mostly average to good quality studies
Vegetarian/Vegan sub-group*	5 RCTs, 1 Case Controlled study	SMD= -0.76 (-1.48 to -0.04), *p*=0.04, *I^2^* =80%	Medium significant effect, considerable heterogeneity	Mostly average to good quality studies
Single food change *	8 RCTs	SMD =-0.43 (-0.76 to -0.10), *p*=0.01, *I^2^*=64%	Small significant effect, substantial heterogeneity	Mostly average to good quality studies
Mediterranean*	1 RCT	SMD =-0.56 (-1.12 to 0.0), *p*=0.05, *I^2^* =n/a	Medium significant effect, heterogeneity n/a (one study)	Average quality study
Elimination diet	4 RCTs	SMD=-0.42 (-0.88 to 0.04), *p*=0.07, *I^2^* =43%	Small non-significant effect, moderate heterogeneity	Average quality studies
Energy and/or macronutrient restriction	5 RCTs	SMD=-0.09 (-0.30 to 0.12), *p*=0.39, *I^2^*=0%	Small non-significant effect, no heterogeneity	Mostly average to good quality studies
Omega 3 focus	1 RCT	SMD=-0.36 (-0.80 to 0.08), *p*=0.11, *I^2^* =n/a	Small non-significant effect, heterogeneity n/a (one study)	Average quality study
**Prego-Dominguez et al.^11^**				
PUFA supplementation (all types) *	46 RCTs	SMD =-0.40 (-0.58 to -0.22), *p*=0.001, *I^2^* =81%	Small significant effect, considerable heterogeneity	Mostly high-quality studies
Omega 3 (from fish oil) *	27 RCTs	SMD= -0.47, (-0.68 to -0.26), *p*=0.001, *I^2^* =77%	Small significant effect, considerable heterogeneity	Unknown
Gammalinolenic acid (Omega-6)	9 RCTs	SMD -0.16 (-0.44 to 0.12), *p*=0.02, *I^2^* =56%	No significant effect, moderate heterogeneity	Unknown
Combined PUFA	3 RCTs	SMD =-0.61 (-1.83 to 0.60), *p*=0.001, *I^2^* =90%	Moderate non-significant effect, considerable heterogeneity	Unknown
Dietary Intervention	5 RCTs	SMD=-0.63 (-1.30 to 0.05), *p*=0.001, *I^2^* =87%	Moderate non-significant effect, considerable heterogeneity	Unknown
