# Supplementary material for: Pain and Diet: A summary of the evidence for the role of diet modification in chronic pain
Source: Br J Pain. Author manuscript; Available in PMC 2024 Feb 21. (PMC10851886; doi:10.1177/20494637231203856)
Supplement: Supplementary file 1 [file EMS192597-supplement-Supplementary_file_1.docx]

**Supplementary file 1**

**Methods of Crawford et al.^9^, Field at al.^10^, Prego-Dominguez et al.^11^**

The core inclusion criteria for all three reviews were studies of adults with chronic pain who received a dietary intervention for at least 2 weeks or more and were assessed primarily with a pain outcome (see Table 2. for the full list of Population, Intervention, Control, Outcomes (PICO) components).  Secondary outcomes were not explored within this commentary. Crawford et al.^9^ utilised systematic reviews and randomised controlled trials (RCTs); a minimum of three were required for analysis of each dietary ingredient, Field et al.^10^ included RCTs and longitudinal pre-post intervention studies, excluding observational studies, and Prego-Dominguez et al.^11^ utilised both experimental and observational study designs.

| Table 2: Population, Intervention, Control, Outcomes (PICO) components | | | |
| --- | --- | --- | --- |
| Component | Crawford et al.^9^ | Field at al.^10^ | Prego-Dominguez et al.^11^ |
| Population | Adults presenting with chronic musculoskeletal pain conditions (including nerve pain). Headache and pain from another injury were excluded. | Adults presenting with chronic pain involving the musculoskeletal or nervous systems. Headache trials and non-musculoskeletal pain such as gastrointestinal or reproductive pain was excluded. | Adults with chronic pain. Childhood or acute pain were excluded. |
| Intervention | Any single or combination of dietary ingredient(s) containing a vitamin, mineral, herb, or other botanical, an amino acid, a concentrate, metabolite, constituent, extract or combination thereof, administered in any form (tablet, liquid etc.).  Any conventional food or sole item of a meal was excluded. | A diet involving whole food or drink change(s) that could be considered part of a common diet and lasted 2 weeks or more.  Medical food, commercial meal replacements, extracts and concentrated nutraceuticals (supplements) were excluded. | PUFA intake or supplementation.  Studies that did not specify the type of fatty acid were excluded. |
| Control | Sham (placebo)/ no treatment. | Characteristics of included studies table included the following comparator groups: placebo, habitual diets, information on healthy eating, lower quantity of or alternative dietary intervention, and N/A. | Characteristics of included studies table included the following comparison groups: Placebo, normal diet, alternative dietary ingredient such as olive or sunflower oil, low Omega-6 diet. |
| Outcomes  (primary) | A continuous outcome measure for pain reduction, at a timepoint closest to three months. | Pain severity or pain interference with function. | Chronic pain according to the definition of the International Association for the Study of Pain. |

All three studies were judged to be methodologically robust, using the Joanna Briggs Institute (JBI) critical appraisal checklist for systematic reviews and research syntheses^13^. The only areas of concern were: the criteria for appraising observational studies^11^ (irrelevant to our analysis due to inclusion of intervention studies only); uncertainty over the number of reviewers who undertook critical appraisal^10^ and no description of publication bias assessment^(9-10)^. Table 3 features the JBI critical appraisal and the corresponding methods for all three systematic reviews.

| Table 3: JBI critical appraisal of Crawford et al. ^9^, Field et al.^10^ and Prego-Dominguez et al.^11^ | | | |
| --- | --- | --- | --- |
| JBI question | Crawford et al.^9^ | Field at al.^10^ | Prego-Dominguez et al.^11^ |
| Is the review question clearly and explicitly stated? | Yes:  ‘Are there dietary supplements/ingredients that can safely mitigate chronic pain in adults with musculoskeletal disorders?’. | Yes:  ‘To identify, compare, and evaluate the published evidence on dietary interventions involving whole-food change(s) for chronic pain management in human participants and to assess the clinical outcome for pain (primary measure)’. | Yes:  ‘To assess whether polyunsaturated fatty acids intake is useful as a preventive or curative tool in chronic pain’. |
| Were the inclusion criteria appropriate for the review question? | Yes:  PICO structure was followed according to question. | Yes:  PICO structure was followed according to question. | Yes:  PICO structure was followed according to question. |
| Was the search strategy appropriate? | Yes:  Search strategy clearly described using PICO components for initial process of a scoping review, followed by focussed review. English language publications only from inception to August 2016. | Yes:  Clear search strategy that addressed each of the identifiable PICO components of the review question.  Two structured searches completed from commencement to December 31, 2019 with no language restrictions. | Yes:  Clear search strategy that addressed each of the identifiable PICO components of the review question.    Studies in any language, searched from inception to May 2015. |
| Were the sources and resources used to search for studies adequate? | Yes:  PubMed, CINAHL, Embase, PsyInfo, MEDLINE | Yes:  MEDLINE, PreMEDLINE, EMBASE, Cochrane library, PschINFO, AMED, CINAHL and Web of Science, reference lists and citation searches. | Yes:  MEDLINE, EMBASE, 5 regional databases of the World Health Organization, Conference Proceedings Citation Index, open access dissertation database, reference lists. |
| Were the criteria for appraising studies appropriate? | Yes:  RCTs: Methodological quality and risk of bias were assessed by using the Scottish Intercollegiate Guidelines Network (SIGN 50) checklist.  Overall quality of evidence and confidence in the effect estimates was assessed using Grading of Recommendations, Assessment, Development and Evaluation (GRADE) with ratings of very low, low, moderate or high quality. | Yes:  Risk of bias (ROB) and study quality were assessed using the National Institutes of Health (NIH) assessment tool for controlled or pre-post interventions.  A quality rating of good (low bias), average (medium bias) or poor (high bias) was given for controlled studies and pre-post studies. | No:  Observational studies were not critically appraised due to small number and no inclusion in meta-analysis.    Yes: Clinical trials were quality assessed using the Jadad index, which rates randomisation, blinding and withdrawals.  A score of 3 or more out of 5 was rated ‘low risk of bias’ and interpreted as ‘high quality’. Score of less than 3 were interpreted as low quality. |
| Was critical appraisal conducted by two or more reviewers independently? | Yes:  Four reviewers independently performed methodological quality assessment of RCTs and two reviewers evaluated systematic reviews. | Unclear:  No explicit description of how many reviewers undertook critical appraisal. | Yes: Two reviewers independently performed quality rating. |
| Were there methods to minimize errors in data extraction? | Yes:  Four reviewers independently performed data extraction. | Yes:  Data were extracted by one reviewer and checked for accuracy by another author. | Yes:  Two reviewers independently performed data extraction using a structured questionnaire. |
| Were the methods used to combine studies appropriate? | Yes:  A meta-analysis was undertaken using a random effects model. | Yes:  A meta-analysis was performed using a random effects model with studies of similar dietary interventions. | Yes:  General meta-analysis was undertaken using both fixed and random effects models. |
| Was the likelihood of publication bias assessed? | No:  Publication bias included as an element of GRADE for individual studies but no overall assessment reported. | No:  Publication bias not reported. | Yes:  Funnel plots and Egger’s regression test were used to detect publication bias.  The trim and fill method was used to correct for potential publication bias. |
| Were recommendations for policy and/or practice supported by the reported data? | Yes:  Recommendations made on benefit and no negative impact. | N/A:  No recommendations for practice were made. | N/A:  No recommendations for practice were made. |
| Were the specific directives for new research appropriate? | Yes:  Hierarchical ranking of recommended areas of research based on effect and quality of evidence. | Yes:  The effect of diet on physiology related to pain to elucidate which pain populations may benefit and the most effective dietary intervention. | Yes:  The dose-response relationship in clinical trials and assessment of PUFAs preventive effects. |

**Results**

Estimates of effectiveness for pain reduction on each dietary intervention within the three reviews are in Table 1, including number/type of studies and assessment of bias. Effect sizes for pain reduction are reported as a standardised mean difference (SMD). In two reviews^(9,11)^, Interpretations of SMD are reported as small (0.2), moderate (0.5) or large (0.8)^14^ with a significance level of *p*=<0.05, a definition we have applied to all three reviews in the commentary. Heterogeneity is reported across all three reviews using the *I^2^* statistic and interpreted as 0-40% (might not be important), 30-60% (may represent moderate), 50-90 (may represent substantial), 75-100% (considerable)^15^.

Taking each review in turn, Crawford et al.^9^ identified 19 dietary ingredients with sufficient evidence for meta-analysis using a pain reduction outcome measure at a timepoint closest to three months. Outcome measures were most commonly the Visual Analogue Scale (VAS) or Western Ontario and McMaster Universities Osteoarthritis Index (WOMAC). Participant characteristics were not reported. The meta-analysis identified that capsaicin, ginger, and rosehip showed small to medium positive effects in pain reduction at a significant level when compared to placebo, based on moderate to high quality evidence. Large effect sizes were found at a significant level for boswellia and curcuma, however these were based on low to very low-quality evidence. Medium effect sizes that reached significance were identified for vitamin D and pycnogenol based on low-quality evidence. Small effect sizes that did not reach significance were found for avocado soybean unsaponifiables, glucosamine plus chondroitin, collagen derivatives and willow bark extract based on mostly low-quality evidence. Findings for PUFA supplementation were taken from Prego-Dominguez et al.^11^ and are reported separately. Meta-analysis was not feasible for the following dietary ingredients due to heterogeneity of treatment, data or outcomes and limited or missing data: Creatine, Devil’s Claw, L-Cartinine, Melatonin, Methylsulfonylmethane, S-adenosyl-L-methionine, and Vitamin E.

Field et al.^10^ identified 43 eligible studies (48 intervention groups) receiving a whole foods dietary intervention. Study durations ranged from 2 weeks to 2 years and change in pain scores were reported from baseline to end of intervention, most commonly with the VAS followed by pain score subsets from WOMAC and SF36. Most participants were female (81%) with an average age of 53 years old. The meta-analysis included 23 controlled studies (25 intervention groups). Comparator groups varied (see Table 2.). The authors identified a consistently small but positive significant effect of whole food dietary change on pain reduction outcomes based on studies of mostly average to good quality and substantial heterogeneity. It was not possible to identify a single diet that stood out for effectiveness.  The vegetarian/vegan, single food change and Mediterranean sub-groups had small to medium effects on pain reduction that reached statistical significance, suggesting a range of whole food dietary change may be helpful in improving chronic pain presentations.   Small but non-significant effects were found for elimination diets, energy or macronutrient restriction and an Omega 3 focus based on mostly average quality studies.

Prego-Domiguez et al.^11^ Identified 51 studies on PUFA supplementation of which 46 studies were suitable for meta-analysis. Intervention periods lasted from 4 weeks to 12 months and pain outcomes were measured using VAS or composite scores for specific pain syndromes. Participant characteristics were not reported. The authors identified that PUFA supplementation (all types) was associated with a meaningful reduction in the risk of chronic pain based on studies of mostly low bias (interpreted as high quality). Sub-analysis by type of fatty acid identified that only Omega-3 showed a significant association with pain; Omega-6 supplementation showed no effect. Combined PUFA and dietary intervention were only significant using a fixed effects model. A further sub-group analysis showed that when the dosage was lower (daily intake ≤ 1.35g) the effect size was higher (moderate effect, SMD -0.55, -0.79 to -0.30) than for the high dose group (small effect, SMD -0.29, -0.56 to -0.03). Similarly, if the intervention period was 3 months or less, there was a larger effect size (moderate effect, SMD -0.56, -0.86 to -0.25), compared to a period of more than 3 months (small effect, SMD -0.24, -0.43 to -0.06).

**Critical appraisal of Crawford et al. ^9^, Field at al.^10^, Prego-Dominguez et al.^11^**

Using the JBI critical appraisal checklist for systematic reviews and research syntheses^13^, the three reviews overall can be considered to provide an adequate and comprehensive summary of evidence relating to chronic pain and diet (see Table 3). It is important to consider however that the reviews of whole food dietary change^10^ and PUFA supplementation^11^ are based on studies of substantial to considerable heterogeneity and so caution should be applied to the generalisability of these findings. The review of dietary ingredients^9^ accounted for heterogeneity using the GRADE approach which implies greater confidence in the ratings of moderate to high certainty evidence.
